# *De novo* transcriptome analyses of host-fungal interactions in oil palm (*Elaeis guineensis* Jacq.)

**DOI:** 10.1186/s12864-016-2368-0

**Published:** 2016-01-19

**Authors:** Chai-Ling Ho, Yung-Chie Tan, Keat-Ai Yeoh, Ahmad-Kamal Ghazali, Wai-Yan Yee, Chee-Choong Hoh

**Affiliations:** Faculty of Biotechnology and Biomolecular Sciences, Universiti Putra Malaysia, 43400 UPM-Serdang, Selangor, Malaysia; Institute of Tropical Agriculture, Universiti Putra Malaysia, 43400 UPM-Serdang, Selangor, Malaysia; Codon Genomics S/B, 26, Jalan Dutamas 7, Taman Dutamas Balakong, 43200 Seri Kembangan, Selangor, Malaysia

## Abstract

**Background:**

Basal stem rot (BSR) is a fungal disease in oil palm (*Elaeis guineensis* Jacq.) which is caused by hemibiotrophic white rot fungi belonging to the *Ganoderma* genus. Molecular responses of oil palm to these pathogens are not well known although this information is crucial to strategize effective measures to eradicate BSR. In order to elucidate the molecular interactions between oil palm and *G. boninense* and its biocontrol fungus *Trichoderma harzianum,* we compared the root transcriptomes of untreated oil palm seedlings with those inoculated with *G. boninense* and *T. harzianum*, respectively.

**Results:**

Differential gene expression analyses revealed that jasmonate (JA) and salicylate (SA) may act in an antagonistic manner in affecting the hormone biosynthesis, signaling, and downstream defense responses in *G. boninense*-treated oil palm roots. In addition, *G. boninense* may compete with the host to control disease symptom through the transcriptional regulation of ethylene (ET) biosynthesis, reactive oxygen species (ROS) production and scavenging. The strengthening of host cell walls and production of pathogenesis-related proteins as well as antifungal secondary metabolites in host plants, are among the important defense mechanisms deployed by oil palm against *G. boninense*. Meanwhile, endophytic *T. harzianum* was shown to improve the of nutrition status and nutrient transportation in host plants.

**Conclusion:**

The findings of this analysis have enhanced our understanding on the molecular interactions of *G. boninense* and oil palm, and also the biocontrol mechanisms involving *T. harzianum*, thus contributing to future formulations of better strategies for prevention and treatment of BSR.

**Electronic supplementary material:**

The online version of this article (doi:10.1186/s12864-016-2368-0) contains supplementary material, which is available to authorized users.

## Background

Basal stem rot (BSR) is a serious and prevalent fungal disease in oil palm that causes significant economic loss to oil palm plantations by reducing oil yield of infected palms [1, 2], causing loss of stands and shortening replanting cycle of new palms [1, 2]. BSR is caused by *Ganoderma* spp. which are able to degrade plant cell wall components including lignin, and cause white rot to oil palm [3]. In advanced infection stage, fruiting bodies may form on oil palm trunks before the rotted infected palms eventually collapse. The disease symptoms of BSR at the foliar part of oil palm include stunted and unopened spear leaves, yellowing and senescence of upper fronds, reduced and “one-sided mottling” canopy [2, 4].

In recent years, fungal DNA-, biochemical-, sensor- and geographic information system -based screening methods for *Ganoderma* spp. in oil palms have been developed [5–8] for early detection of BSR. The mitigation actions practiced by oil palm plantations include sanitation before replanting, isolation of infected or diseased palms by elimination and burning of infected palms [9], and application of fungicides [10]. The application of beneficial microbes as biocontrol agents such as *Trichoderma* spp., mycorrhizae and plant growth promoting bacteria have also been demonstrated to be able to alleviate BSR incidences [11–13].

Oil palm lines that are tolerant to *Ganoderma* spp. have been reported previously [14, 15]. However, the genes that are responsible for their tolerance are unknown. Genes and gene products that contribute to the virulence of *Ganoderma* spp., and defense response of oil palm to these pathogens are not well known although this information is crucial to strategize effective measures against the devastating disease.

In recent years, transcript sequences encoding pathogenesis related (PR) proteins such as chitinases, glucanases, defensins, protease inhibitors [16–19], and proteins that are potentially involved in oil palm defense [17, 20, 21] have been reported. However, the information is partial and insufficient to delineate a complete picture of oil palm defense in response to *Ganoderma* spp. due to a limited number of transcripts and proteins being analyzed.

The main objective of this study was to elucidate the molecular responses of oil palm roots colonized by *G. boninense* by comparing the transcriptomes of inoculated and uninoculated oil palm seedlings with this pathogenic fungus. In addition, the fungal transcripts that were present in the transcriptome of treated oil palm roots were identified to lend insight into the infection mechanisms of *G. boninense*. We also compared the differentially expressed genes (DEGs) in oil palm roots upon treatments with *G. boninense* and *T. harzianum*, respectively, to shed light on different responses of oil palm roots to pathogenic and beneficial fungi, and to provide evidence supporting the biocontrol mechanisms proposed for *T. harzianum* against *G. boninense*. A comprehensive understanding of oil palm defense responses opens up opportunities for developing strategies to eradicate the disease and to enhance the disease tolerance/resistance of this important crop, noting that it is one of the major producers of edible oil and oil related products in the world.

## Results

### Transcriptomes of oil palm roots in response to *G. boninense* and *T. harzianum*

In this experiment, we aimed to identify the genes that are involved in the molecular responses of oil palm roots to the artificial inoculation of *G. boninense* and *T. harzianum*. The G-treated oil palm root tissues (whole root sections) collected were not separated based on duration of inoculation due to the non-synchronous nature of infection of *G. boninense* on the roots from the same plant at a particular time point. During the course of *Ganoderma*(G)-treatment, lesions and mycelia were observed on the root surface of G-treated seedlings at 6 and 12 weeks post inoculation (wpi), indicating that *G. boninense* has successfully colonized the roots. However, no other disease symptoms such as yellowing of leaves and formation of fruiting bodies were observed. The mRNA samples of oil palm roots from nine biological replicates in G-treatment from 3–12 wpi, covering infection stages from recognition of pathogen to tissue necrosis, were pooled and sequenced to reveal the transcripts involved. The roots from an equal number of biological replicates of untreated oil palms were also sampled from 3–12 wpi, for mRNA isolation and sequencing. In total, 111,832 transcripts were assembled from 37,784,896 processed and filtered reads (approximately 2.79 × 10^9^ bases) obtained from mRNA-seq analysis of untreated roots compared to 50,025 transcripts assembled from 14,495,512 processed and filtered reads (approximately 5.45 × 10^8^ bases) which were obtained from mRNA-seq analysis of G-treated roots.

In addition, the transcriptome of oil palm inoculated with *T. harzianum* was also profiled. Throughout the 12 week-treatment, the spore counts of *T. harzianum* in the soil around the *Trichoderma* (T)-treated seedlings were maintained above 10^4^ colonies per unit (cfu) per g of soil to ensure the treatment was effective while the spore counts in the untreated soil were estimated to be 10^2^ cfu per g of soil. The mRNA samples from oil palm roots in T-treatment, from 3–12 wpi were pooled and sequenced. The processed and filtered reads (16,665,379 reads which total up to approximately 6.33 x 10^8^ bases) were obtained from mRNA-seq analysis of T-treated roots and assembled into 45,968 transcripts.

The assembled sequences (transcripts) obtained from untreated, G- and T-treated oil palm roots were then clustered to reduce the number of overlapping sequences in each transcriptome for functional annotation. Approximately 50 % of the assembled sequences (unigenes) have significant matches (E values ≤10^−5^) to the non-redundant protein database at National Center for Biotechnology Information (NCBI). About 84 % of these unigenes which have significant matches to protein encoding sequences from Viridiplantae were used for identification of differentially expressed genes (DEGs) in pairwise samples.

Biological averaging where pooled biological replicates instead of individual biological replicates was employed in DEG analysis in this study. Although the results in this study may not provide the same statistical resolution by replicated mathematical averaged experiments, biological averaging for DEG analysis is neither new nor unprecedented in both cDNA microarray and mRNA-seq [22–28]. Meanwhile, DEG analysis by DESeq [29] has been reported to be more conservative and predicted less number of DEGs in comparison with another method which has been developed for mRNA-seq analysis without replicates [22]. In this study, the variance was estimated by treating the samples being compared as if they were replicates of the same condition. We noted that without individual biological replicates, the statistical significance could only represent differences in G- or T-treated sample and untreated sample being compared. Nevertheless, the gene expression measurements represented averages across nine oil palm seedlings since roots from nine oil palm seedlings were pooled to generate the sequencing libraries.

The expression levels of DEGs in 21 pairwise samples were further verified by quantitative reverse-transcription (qRT)-PCR (Table 1) on the same RNA samples used for sequencing, and approximately 86 % of them support the results predicted *in silico* whereby most of the genes were shown to have consistent expression patterns (up- or down-regulation) by both DEG analysis and qRT-PCR*.* We noted differences in the magnitude of gene expression measured by these two approaches which are most probably caused by differences in sensitivity and normalization methods, however the gene expression patterns measured by both approaches are largely similar.

**Table 1:** Verification of DEGs using real-time qRT-PCR

Unigene	Putative identity	Fold change of expression using	
		Transcriptome sequencing	Real-time qRT-PCR
		In G-treated oil palm seedlings compared to untreated seedlings	
15029	1-Aminocyclopropane-1-carboxylate oxidase	107	4.84±0.43
13913^a^	1-Aminocyclopropane-1-carboxylate oxidase	−2.70	1.01±0.03
21170	Chitinase	12.5	3.59±0.58
21171	Chitinase	16.8	2.93±0.84
21174	Chitinase Class III	10.1	4.03±0.91
595	Early flowering protein	5.85	3.25±0.66
23670	β-1,3-Glucanase	6.17	9.18±0.63
4320^a^	NBS-LRR-resistance protein	−10.1	1.88±0.16
152	Pathogenesis-related protein	20.4	4.84±0.36
23132	Pathogenesis-related protein 4	8.40	3.03±0.54
15545	Type 2 ribosome-inactivating protein precursor	9.26	2.39±0.34
15323	Thaumatin-like protein	17.5	2.29±0.16
28483	Predicted protein	2.54	2.29±0.15
28249	Hypersensitive induced reaction1	6.37	1.45±0.16
18366	Hypothetical protein SORBIDRAFT 02g042630	8.76	1.09±0.13
18282^a^	Fiber protein Fb2	−1.12	1.25±0.08
308	Ethylene insensitive-like protein 4	1.26	1.61±0.11
		In T-treated oil palm seedlings compared to untreated seedlings	
13913	1-Aminocyclopropane-1-carboxylate oxidase	−12.9	−1.82±0.04
21170	Chitinase	8.47	3.85±0.95
4320	NBS-LRR-resistance protein	10.4	3.21±0.48
25702	Nitrate reductase 1	9.46	2.19±0.24

^a^Genes that show discrepancies in their gene expression patterns when measured by transcriptome sequencing and real-time qRT-PCR

### DEGs of oil palm roots during *Ganoderma* colonization

In total, 1,996 and 1,219 oil palm genes were found to be up- and down-regulated 4-fold and more in G-treated oil palm roots, respectively (Additional file 1: Table S1) compared to the untreated oil palm roots. These oil palm genes could either form part of the host defense against fungal invasion, or a consequence of the suppression of host defense system by the pathogen upon G-treatment. The up-regulated genes in oil palm treated with G. *boninense* were found to be enriched in 85 Gene Ontology (GO) Slim terms: 40 in biological process (BP), 33 in molecular function (MF), and 12 in cellular compartment (CC) (Table 2). Among these GOSlim terms are those related to defense response, cellular response to salicylic acid (SA) stimulus, nitric oxide mediated signal transduction, 1-aminocyclopropane-1-carboxylate (ACC) oxidase activity, naringenin-chalcone synthase and others (Table 2). On the other hand, the down-regulated DEGs were found to be enriched in 27 GOSlim terms (8 BP, 6 MF, 13 CC; Table 2). The enriched GOSlim terms among the down-regulated DEGs in G-treated oil palm roots include those related to generation of precursor metabolites and energy, photosynthesis, electron transport chain, ribosome biogenesis, xyloglucan:xyloglucosyl transferase activity and others (Table 2).

**Table 2:** Summary of GO terms that are enriched in DEGs of G-treated oil palm roots compared to untreated roots

GO terms that are enriched in DEGs of G-treated oil palm roots compared to untreated oil palm roots			
Biological process			
Up-regulated genes			
In G-treatment only			
GO:0030636	acetate derivative biosynthetic process	GO:0046483	heterocycle metabolic process
GO:0030635	acetate derivative metabolic process	GO:0006972	hyperosmotic response
GO:0006083	acetate metabolic process	GO:0006811	ion transport
GO:0006026	aminoglycan catabolic process	GO:0006629	lipid metabolic process
GO:0006022	aminoglycan metabolic process	GO:0051179	localization
GO:0019438	aromatic compound biosynthetic process	GO:0007263	nitric oxide mediated signal transduction
GO:0015976	carbon utilization	GO:0071704	organic substance metabolic process
GO:0016052	carbohydrate catabolic process	GO:0015846	polyamine transport
GO:0005975	carbohydrate metabolic process	GO:0009152	purine ribonucleotide biosynthetic process
GO:0015977	carbon fixation	GO:0032268	regulation of cellular protein metabolic process
GO:0071281	cellular response to iron ion	GO:0010035	response to inorganic substance
GO:0071446	cellular response to salicylic acid stimulus	GO:0010039	response to iron ion
GO:0009805	coumarin biosynthetic process	GO:0019748	secondary metabolic process
GO:0006952	defense response	GO:0001736	establishment of planar polarity
GO:0006097	glyoxylate cycle	GO:0006099	tricarboxylic acid cycle
In both G- and T-treatments			
GO:0043450	alkene biosynthetic process	GO:0009056	catabolic process
GO:0009058	biosynthetic process	GO:0006091	generation of precursor metabolites and energy
GO:0055114	oxidation-reduction process	GO:0010817	regulation of hormone levels
GO:0080167	response to karrikin	GO:0051246	regulation of protein metabolic process
GO:0044283	small molecule biosynthetic process	GO:0006414	translational elongation
In T-treatment only			
GO:0015837	amine transport	GO:0044242	cellular lipid catabolic process
GO:0046942	carboxylic acid transport	GO:0006732	coenzyme metabolic process
GO:0044249	cellular biosynthetic process	GO:0006006	glucose metabolic process
GO:0034754	cellular hormone metabolic process	GO:0071705	nitrogen compound transport
Down-regulated genes			
In G-treatment only			
GO:0071103	DNA conformation change	GO:0015979	photosynthesis
GO:0022900	electron transport chain	GO:0006412	translation
In both G- and T-treatments			
GO:0006091	generation of precursor metabolites and energy	GO:0015992	proton transport
GO:0006818	hydrogen transport	GO:0042254	ribosome biogenesis
In T-treatment only			
GO:0006119	oxidative phosphorylation	GO:0010200	response to chitin
Cellular compartment			
Up-regulated genes			
In G-treatment only			
GO:0009341	beta-galactosidase complex	GO:0005887	integral to plasma membrane
GO:0071944	cell periphery	GO:0016020	membrane
GO:0005618	cell wall	GO:0030117	membrane coat
GO:0048475	coated membrane	GO:0044425	membrane part
GO:0030312	external encapsulating structure	GO:0005773	vacuole
GO:0030660	Golgi-associated vesicle membrane		
In both G- and T-treatments			
GO:0009325	nitrate reductase complex		
Down-regulated genes			
In G-treatment only			
GO:0044429	mitochondrial part	GO:0032993	protein-DNA complex
GO:0043228	non-membrane-bounded organelle	GO:0045259	proton-transporting ATP synthase complex
GO:0000786	nucleosome	GO:0016469	proton-transporting two-sector ATPase complex
GO:0031984	organelle subcompartment	GO:0009579	thylakoid
GO:0034357	photosynthetic membrane	GO:0042716	plasma membrane-derived chromatophore
GO:0030077	plasma membrane light-harvesting complex		
In both G- and T-treatments			
GO:0030529	ribonucleoprotein complex	GO:0005840	ribosome
Molecular function			
Up-regulated genes			
In G-treatment only			
GO:0009815	1-aminocyclopropane-1-carboxylate oxidase activity	GO:0016829	lyase activity
GO:0004013	adenosylhomocysteinase activity	GO:0003796	lysozyme activity
GO:0030246	carbohydrate binding	GO:0000287	magnesium ion binding
GO:0003824	catalytic activity	GO:0046872	metal ion binding
GO:0016206	catechol O-methyltransferase activity	GO:0016210	naringenin-chalcone synthase activity
GO:0043169	cation binding	GO:0016491	oxidoreductase activity
GO:0016405	CoA-ligase activity	GO:0016903	oxidoreductase activity, acting on the aldehyde or oxo group of donors
GO:0016798	hydrolase activity, acting on glycosyl bonds	GO:0016616	oxidoreductase activity, acting on the CH-OH group of donors, NAD or NADP as acceptor
GO:0005222	intracellular cAMP activated cation channel activity	GO:0030414	peptidase inhibitor activity
GO:0043167	ion binding	GO:0004665	prephenate dehydrogenase (NADP+) activity
GO:0005506	iron ion binding	GO:0022857	transmembrane transporter activity
GO:0052633	isocitrate hydro-lyase (cis-aconitate-forming) activity	GO:0005215	transporter activity
GO:0016874	ligase activity	GO:0016877	ligase activity, forming carbon-sulfur bonds
In both G- and T-treatments			
GO:0005275	amine transmembrane transporter activity	GO:0009703	nitrate reductase (NADH) activity
GO:0003680	AT DNA binding	GO:0016614	oxidoreductase activity, acting on CH-OH group of donors
GO:0004108	citrate (Si)-synthase activity	GO:0050242	pyruvate, phosphate dikinase activity
In T-treatment only			
GO:0004022	alcohol dehydrogenase (NAD) activity	GO:0005342	organic acid transmembrane transporter activity
GO:0015174	basic amino acid transmembrane transporter activity	GO:0008756	o-succinylbenzoate-CoA ligase activity
GO:0020037	heme binding	GO:0016661	oxidoreductase activity, acting on other nitrogenous compounds as donors
GO:0009678	hydrogen-translocating pyrophosphatase activity	GO:0030598	rRNA N-glycosylase activity
GO:0004427	inorganic diphosphatase activity	GO:0046906	tetrapyrrole binding
Down-regulated genes			
In G-treatment only			
GO:0004767	sphingomyelin phosphodiesterase activity	GO:0016762	xyloglucan:xyloglucosyl transferase activity
In both G- and T-treatments			
GO:0045158	electron transporter, transferring electrons within cytochrome b6/f complex of photosystem II activity	GO:0003735	structural constituent of ribosome
GO:0045156	electron transporter, transferring electrons within the cyclic electron transport pathway of photosynthesis activity	GO:0005198	structural molecule activity
In T-treatment only			
GO:0043168	anion binding	GO:0003954	NADH dehydrogenase activity
GO:0004427	inorganic diphosphatase activity	GO:0016651	oxidoreductase activity, acting on NADH or NADPH
GO:0005506	iron ion binding		

**Fig. 1:**
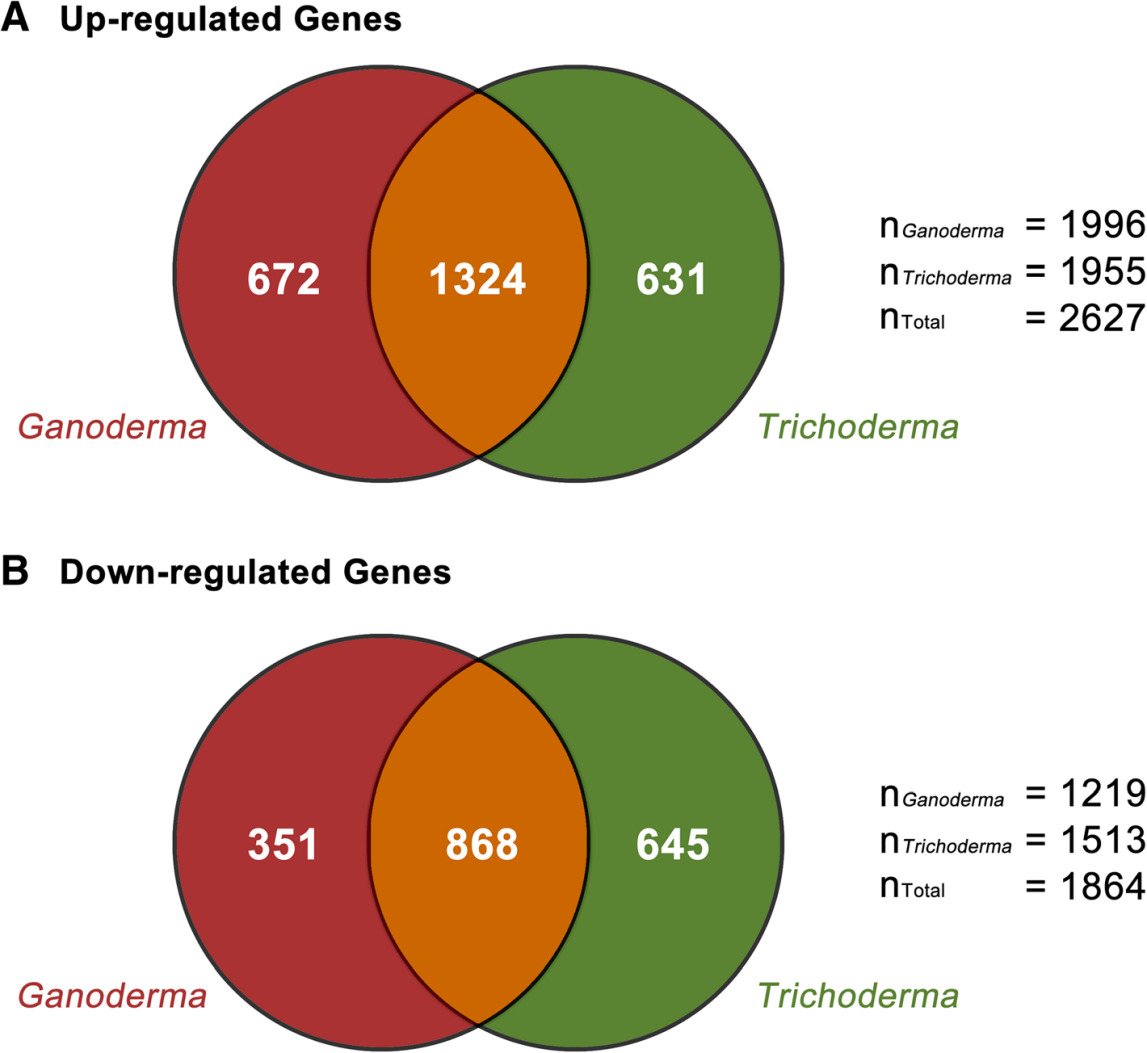
Venn diagrams of differentially expressed genes in G- and T-treated samples compared to the controls. **a** and **b** show the numbers of up-regulated and down-regulated genes in treated samples, respectively. Up-regulated genes include genes with Log_2_(fold change) value ≥ 2, and genes that are present in treated samples but absent in controls; while down-regulated genes include genes with expression Log_2_(fold change) value ≤ −2, and genes that are present in controls but absent in treated samples. The numbers in the red and green regions represent the numbers of up- and down-regulated genes in G- and T-treated samples, respectively. The numbers in the orange region represent the numbers of genes that are differentially expressed in both G- and T-treated samples. Numbers outside the Venn diagram represent the total numbers of up- and down-regulated DEGs by both treatments and each treatment

Among the GOSlim terms that were enriched in the pool of up-regulated genes in G-treated oil palm roots, 17 of them (9 BP, 6 MF, 1 CC) were also found to be enriched among the up-regulated DEGs in T-treated oil palm roots*,* including those related to response to karrikin, generation of precursor metabolites and energy, translational elongation, amine transmembrane transporter activity, nitrate reductase (NADH) activity and others (Table 2). A total of 10 GOSlim terms that were enriched among the down-regulated genes in G-treated oil palm roots (4 BP, 4 MF, 2 CC) were also found to be enriched among the down-regulated DEGs in T-treated oil palm roots, including those related to ribosome biogenesis, electron transport chain, generation of precursor metabolites and energy (Table 2). These GOSlim terms may reflect the general responses of oil palm roots to fungi irrespective of whether they are pathogenic or beneficial, and their modes of infection. True enough, 1,324 and 868 DEGs were up- and down-regulated by both G- and T-treatments while 672 and 351 DEGs were uniquely up- and down-regulated by G-treatment only (Fig. 1).

**Fig. 2:**
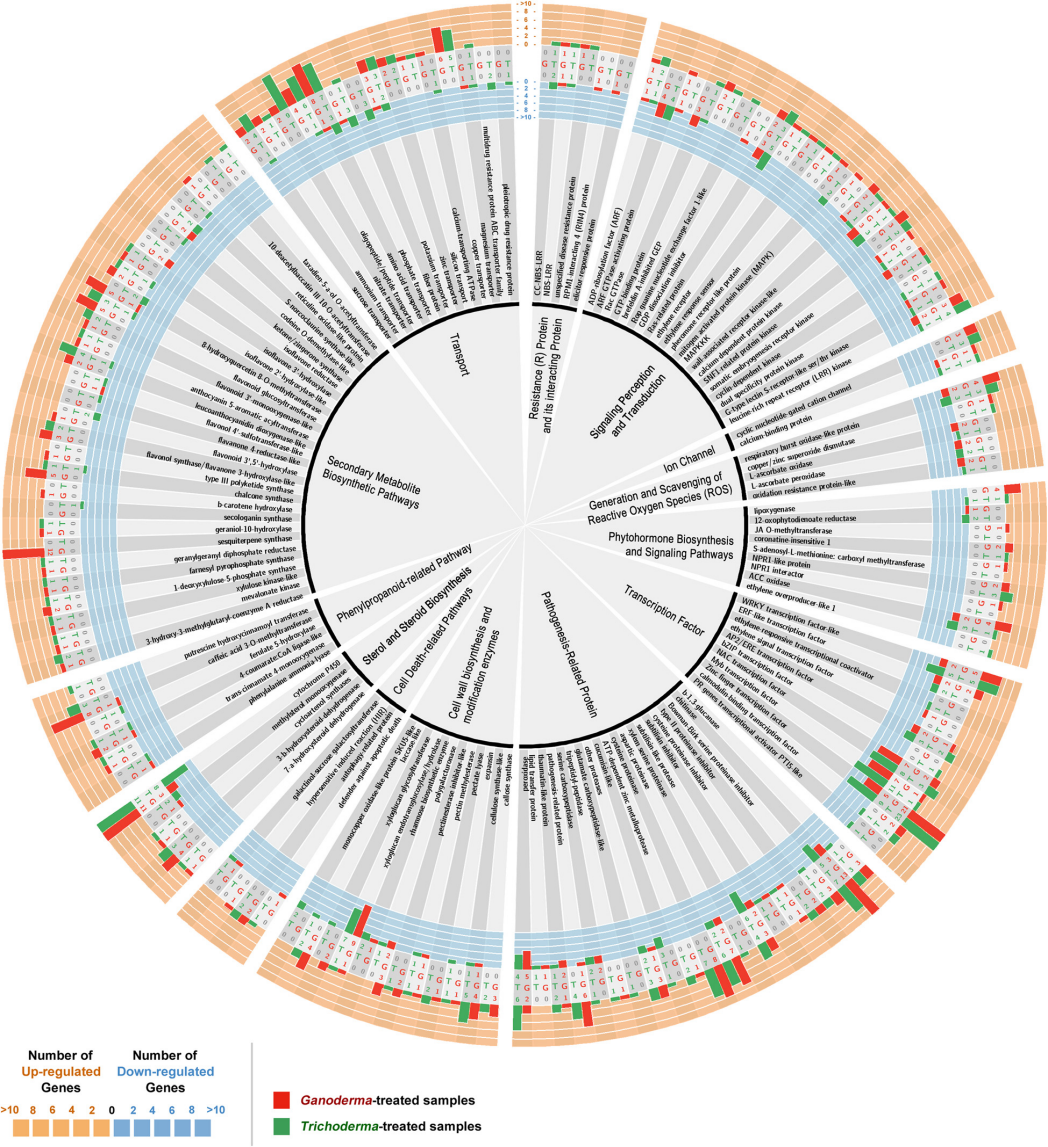
Number of DEGs in the G- and T-treated oil palm roots compared to untreated roots that are related to oil palm defense mechanisms. The most outer ring (in orange color) shows the number of up-regulated DEGs of treated samples while the blue ring shows the number of down-regulated DEGs of treated samples. Histograms and numbers in red and green colors represent the number of DEGs in *Ganoderma* (G)- and *Trichoderma* (T)-treated samples, respectively. The putative functions of DEGs are shown in the two most inner rings. The figure was generated by Circos software [80]

**Fig. 3:**
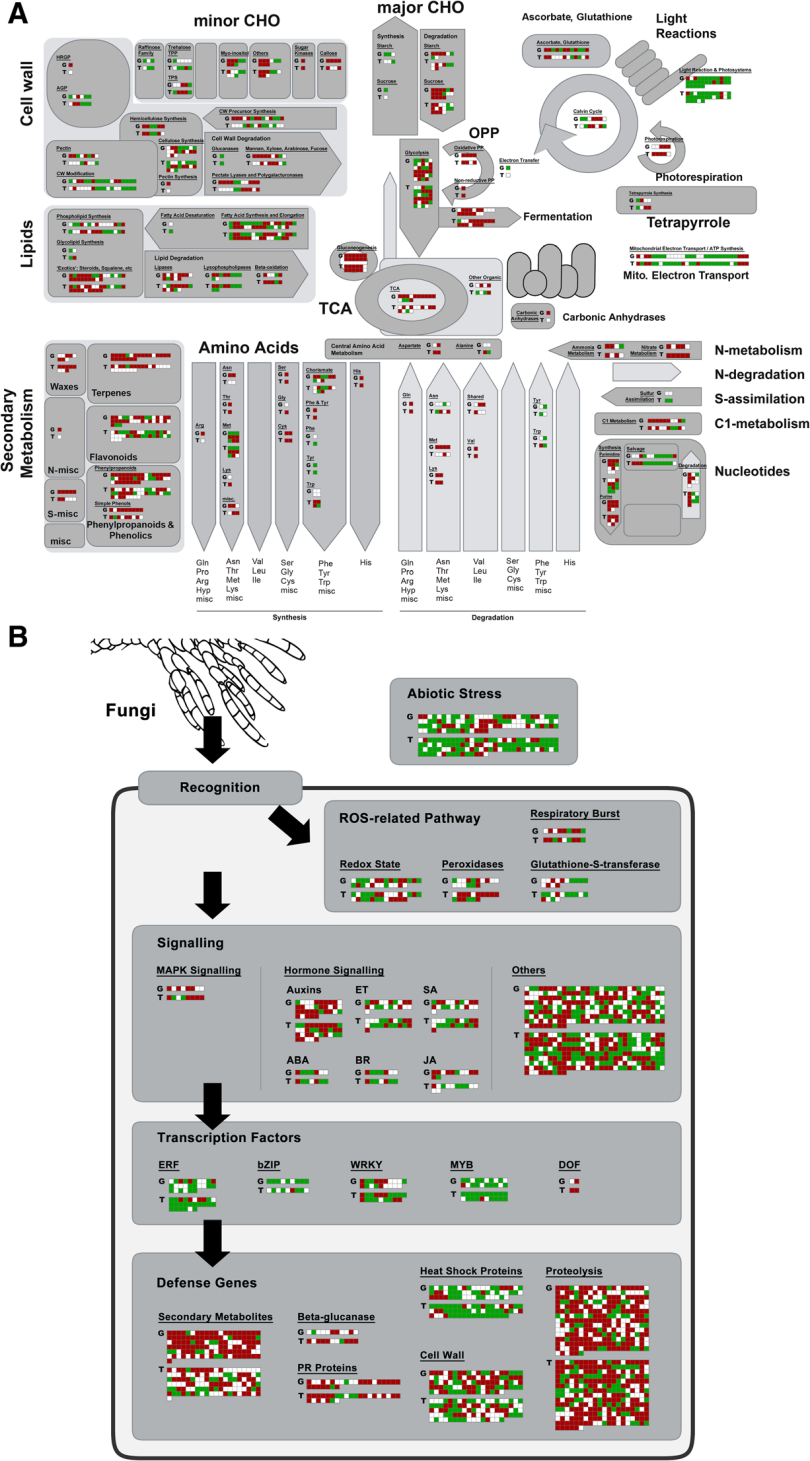
Snapshots of DEGs in the G- and T-treated oil palm roots compared to untreated roots. The snapshots were generated by MapMan software [77] for the main metabolic pathways (**a**), and stress-related pathways (**b**). The panels in red and green represent DEGs (*P-*value < 0.05) with Log_2_(fold change) value ≥ 2 (up-regulation in treated oil palms) and Log_2_(fold change) value ≤ −2 (down-regulation in treated oil palms), respectively. The G and T letters on the left hand side of the panels indicate the DEGs for *Ganoderma* (G)- and *Trichoderma* (T)-treated samples, respectively. White panels represent DEGs with Log_2_(fold change) value between −2 and 2. ABA, abscisic acid; BR, brassinosteroids; CHO, carbohydrates; ET, ethylene; JA, jasmonic acid; Mito., mitochondrial; OPP, oxidative pentose phosphate pathway; PR, pathogenesis-related; ROS, reactive oxygen species; SA, salicylic acid; TCA, tricarboxylic acid

Figure 2 shows the DEGs in G-treated oil palm roots compared to the untreated oil palm roots that are involved in the defense response including signal perception and transduction, phytohormone biosynthesis and signaling, transcription factor, generation and scavenging of reactive oxygen species (ROS), production of secondary metabolites, cell wall biosynthesis and modification enzymes and others. It also compares the number of DEGs in T-treated oil palm seedlings in relative to that of the untreated oil palm seedlings. Snapshots of the DEGs in G- and T-treated oil palm roots over the main metabolic pathways and stress-related pathways are also displayed in Fig. 3.

### DEGs of oil palm roots in response to *T. harzianum*

A total of 1,955 and 1,513 genes were found to be up- and down-regulated 4-fold and more in T-treated oil palm roots, respectively, compared to the untreated oil palm roots (Additional file 1: Table S2). However, only 631 and 645 of these DEGs were found to be uniquely up- and down-regulated by T-treatment only (Fig. 1). Thirty five GOSlim terms (17 BP, 17 MF, 1 CC) were found to be enriched among the up-regulated DEGs in T-treated oil palm roots with 18 of them (8 BP, 10 MF) found to be enriched among the up-regulated DEGs in T-treated oil palm only but not among those in G-treated oil palm roots (Table 2)*.* The majority of these GOSlim terms are related to transportation including amine transport, nitrogen compound transport and carboxylic acid transport (Table 2). On the other hand, the unique down-regulated DEGs in T-treated oil palm roots were found to be enriched in 17 GOSlim terms (6 BP, 9 MF, 2 CC) with only 7 of them being unique to T-treated oil palm roots including response to chitin and anion binding (Table 2).

### Fungal transcripts in treated oil palm roots

The presence of fungal transcripts in G-treated oil palm roots allowed us to have an overview of the genes being expressed in *G. boninense* during its infection of host plant. The fungal transcripts in treated oil palm roots were identified among the unigenes as sequences with high identities to fungal sequences, or protein encoding sequences that showed high identities to those belonging to fungi. The number of reads that showed high identities to those of *Ganoderma* sp. 10597 SS1, *Trichoderma harzianum* CBS226.95, *Trichoderma virens* Gv29-8 and *Trichoderma reesei* were 3.1 %, 0.9 % and 2.4 % of the total mRNA-seq reads obtained from G-, T-treated and untreated oil palm roots, respectively. The number of protein encoding sequences that showed high identities to those belonging to fungi represents 13 % of the total unigenes that have significant matches to the non-redundant protein database at NCBI. The numbers of reads that contribute to these unigenes were 5.8 %, 0.4 % and 0.3 % of the mRNA-seq reads obtained from G-, T-treated and untreated oil palm roots, respectively. Table 3 (and Additional file 1: Table S3) shows the fungal transcripts in treated and untreated oil palm roots. Among these transcripts are those encoding enzymes involved in lignin metabolism such as manganese peroxidase and laccases. Besides, there are a high number of fungal transcripts encoding glycoside hydrolases and glycosyltransferases from various families, carbohydrate binding modules, cellulases and polysaccharide lyase among the fungal transcripts in G-treated oil palm roots. On the other hand, transcripts encoding cell wall degrading/modification enzymes were not present in the transcriptome of T-treated oil palm roots. The genes encoding phosphate transporters, sugar transporter and carbohydrate metabolism were found to be abundant among the fungal transcripts in T-treated oil palm roots (Table 3).

**Table 3:** Fungal transcripts in treated and untreated oil palm seedlings

Putative functions of sequence	Total number	Number of transcripts in		
		G	T	UT
Pathogenesis				
Fungal allergen/elicitor	7	5	4	4
immunomodulatory protein	1	1	0	0
salicylate hydroxylase	2	1	1	1
Cell death-related				
apoptosis-linked protein	1	1	0	0
autophagy protein	3	3	2	2
necrosis- and ethylene-inducing protein	1	1	0	0
programmed cell death protein	1	1	0	0
Hydrogen scavenging				
catalase	8	7	0	1
Mn superoxide dismutase	6	5	2	1
peroxiredoxin	5	5	1	1
Cell wall modification and degradation				
α-amylase	3	3	0	0
acetyl xylan esterase	8	5	2	3
α-galactosidase	8	8	0	0
arabinofuranosidase	3	3	0	0
arabinogalactan endo-1,4-β-galactosidase	1	1	0	0
β-glucosidase	9	9	0	0
β-hexosaminidase	2	1	0	1
carbohydrate esterase	15	13	1	2
carbohydrate-binding module	9	6	2	5
cellulase	20	17	7	7
cellulose binding protein	4	3	1	2
copper radical oxidase	9	4	3	4
dye decolorizing peroxidase	3	2	0	1
endo-1,3(4)-β-glucanase	13	3	11	11
endo-1,4-β-glucanase	16	15	3	3
endo-1,4-β-xylanase	10	7	6	7
endo-β-mannanase	1	1	0	0
endo-β-N-acetylglucosaminidase	7	7	0	0
endogalactanase	1	1	0	0
endo-polygalacturonase	1	1	1	0
feruloyl esterase	5	5	1	1
galactan 1,3-β-galactosidase	2	2	0	0
glucan 1,3 β-glucosidase	4	3	2	3
glycoside hydrolase family	186	127	21	36
glycosyltransferase	51	44	9	12
glyoxal oxidase	11	5	8	8
laccase	11	10	0	1
L-rhamnosidase	1	1	0	0
manganese peroxidase	12	9	1	3
pectinesterase	2	2	0	0
polysaccharide lyase	7	7	0	0
polysaccharide synthase	2	2	0	0
rhamnogalacturonase	4	4	0	0
Fatty acid biosynthesis				
3-ketoacyl-CoA thiolase	4	3	0	1
3-methylcrotonyl-CoA carboxylase	2	2	0	0
dihydroceramide δ (4)-desaturase	2	2	0	0
fatty acid desaturase	22	17	8	11
fatty acid elongase	2	2	0	0
fatty acid hydroxylase	2	2	0	1
fatty acid synthase	2	0	1	2
Phytosterol and steroid biosynthesis				
hydroxysteroid dehydrogenase	1	1	0	0
lanosterol 14-α-demethylase	1	1	0	0
mevalonate pyrophosphate decarboxylase	5	5	0	0
sterol 14 α-demethylase	1	1	0	0
sterol 24-C-methyltransferase	2	0	1	1
sterol δ 5,6-desaturase	1	1	0	0
sterol-binding protein	1	1	0	0
Detoxification				
aflatoxin-detoxifizyme	1	1	0	0
dienelactone hydrolase	4	4	0	0
Carbon metabolism				
glycogen phosphorylase	1	1	0	0
glycogen synthase	8	6	2	2
trehalose synthase	5	5	0	0
Nitrogen metabolism				
2-nitropropane dioxygenase	4	2	1	2
NAD(P)H-nitrite reductase	1	1	0	0
nitroreductase	3	3	0	0
Protein degradation				
aspartic peptidase	16	11	2	9
aspartic protease	3	2	0	1
aspartic proteinase	1	1	0	0
endopeptidase	3	3	0	1
metalloproteinase	3	3	1	0
tripeptidyl peptidase	2	2	0	0
Amino acid metabolism				
aspartate aminotransferase	8	7	0	1
glutamate decarboxylase	1	0	0	1
glutamate dehydrogenase	16	15	0	1
glutamine synthetase	14	10	10	11
Urea cycle				
arginase	2	2	0	1
argininosuccinate lyase	2	1	1	2
argininosuccinate synthetase	3	3	0	0
ornithine carbamoyltransferase	1	1	0	0
Nutrient transport				
amino acid permease	15	9	8	8
amino acid transporter	2	2	0	0
ammonium transporter	3	2	2	2
glucose transporter	13	10	8	7
hexose transporter	14	10	11	12
monosaccharide transporter	10	6	7	7
multidrug transporter	8	6	1	2
oligopeptide transporter	17	15	0	2
peptide transporter	4	0	1	4
phosphate transporter	25	14	13	18
phospholipid transporter	2	0	0	2
sterol carrier protein	1	1	0	0
sugar transporter	7	5	2	2
sulfate transporter	2	1	0	2

## Discussion

### What are the genes and biological processes involved in oil palm roots infected by hemibiotrophic *G*. *boninense*?

The DEGs in G-treated oil palm roots in comparison with the untreated oil palm roots enabled us to deduce the proteins and pathways involved in the defense response of host plant in response to *G. boninense*. In the initial stage of infection by *G. boninense*, recognition of pathogen by oil palm could be initiated by the binding of pathogen-associated molecular patterns (PAMPs) from *G. boninense* to pattern recognition receptors (PRRs) residing extracellular or at the plasma membrane. This is evident by the presence of DEGs encoding numerous receptors in the transcriptome of G-treated oil palm roots such as receptor-like kinases (RLKs) and receptor-like proteins (RLPs).

The components of fungal cell wall or cell membrane such as chitin, glucans and ergosterol have been reported as PAMPs in many pathosystems [30]. This is supported by our findings which revealed that various oil palm chitinases and glucanases were indeed up-regulated in oil palm roots in response to G-treatment. These cell wall degrading enzymes (CWDEs) may degrade fungal cell wall, releasing chitins and glucans. Alternatively, ergosterol which was reported to be important for the virulence of pathogen [30] may also elicit PAMP-triggered immunity (PTI) in oil palm. In addition, degraded plant cell wall or cell wall components (also known as damage-associated molecular patterns, DAMPs) such as oligogalacturonates may serve as endogenous elicitors that bind to PRRs [31]. Fungal transcripts encoding enzymes involved in the biosynthesis of ergosterol, and CWDEs such as polygalacturonases, laccases and many other glycosyl hydrolases were found to be in high abundance in G-treated oil palm roots (Table 3).

In addition to PTI, a rapid and robust plant response i.e. effector-triggered immunity (ETI) which is based on highly polymorphic resistance (R) proteins [32] can also be activated in plants upon the recognition of avirulence (Avr) effectors released by biotrophic pathogens. ETI often blocks further invasion of pathogens by inducing a hypersensitive response and other locally induced defence responses which confine pathogens at the infection site and limit their growth and supply of nutrients. So far, only a Toll/interleukin 1 receptor domain R protein has been reported to be associated with the resistance of *Arabidopsis* against the necrotrophic *Leptospharia maculans* [33]. Although it is premature to speculate the presence of R proteins that are associated with the hemibiotrophic *Ganoderma* spp. in oil palm, several oil palm genes encoding R proteins with yet to be known functions were found to be differentially regulated in this study (Fig. 2). Among them, the gene encoding RPM1-interacting protein 4 (RIN4) which is a mediator of recognition of an R protein (RPM1) and also a negative regulator of PTI [34], was found to be down-regulated in G-treated oil palm roots. However, the gene encoding RPM1 was not found to be differentially regulated in G-treated oil palm roots. Its association with the susceptibility of oil palm to *Ganoderma* spp. was not known.

Signaling, ion fluxes, respiratory burst and generation of reactive oxygen species (ROS) may occur in infected roots as evident by the presence of DEGs encoding putative ADP-ribosylation factor (ARF), ARF GTPase activating protein, Rac small GTPases, GTP binding protein, mitogen associated protein kinase (MAPK), calcium dependent protein kinase (CDPK), ion channels, calcium transporters, respiratory burst oxidase (Rboh) and superoxide dismutase (SOD) in G-treated roots. Plasma membrane dynamics play an important role in plant defense whereby the residing calcium and anion channels and associated ion fluxes are important signaling events that regulate host defence. Besides, Rboh or NADPH oxidase which resides in the plant plasma membrane produces superoxide and other ROS including hydrogen peroxide that can cause lipid peroxidation, cross-linking of cell wall proteins and lignification. Hydrogen peroxide can also travel into plant cytoplasm serving as signaling molecule of other defense response including ethylene (ET) biosynthesis and hypersensitive reaction (HR)-associated cell death [35].

Lipid peroxidation of plasma membrane followed by lipoxygenation of fatty acids may lead to the biosynthesis of oxylipins, including 12-oxophytodienoate (OPDA) which is the precursor of jasmonic acid (JA). A few genes encoding lipoxygenases were up-regulated in G-treated oil palm roots which may possibly lead to an increase in oxylipins, however the expression of gene encoding 13-lipoxygenase which is involved in the lipoxygenation of α-linolenic acid leading to the production of 12-OPDA was not differentially expressed in G-treated oil palm roots. Lipoxygenase-catalysed reactions can alternatively produce toxic volatile and non-volatile fatty acid-derived secondary metabolites that can directly attach to pathogens [36]. In addition, the genes encoding 12-OPTA reductase (OPR) and JA O-methyltransferase were down- and up-regulated in G-treated oil palm roots, respectively; indicating that the production of JA could be reduced while the pre-existing JA could be methylated to methyl-JA (an inactivated form of JA and a signal molecule). Taken together, it is plausible that the JA-induced defence against *G. boninense* in G-treated oil palm was down-regulated by the pathogen.

The genes encoding coronatine-insensitive 1 (COI) was up-regulated in G-treated oil palm roots. COI is a component of SCF (COI1) E3 ubiquitin ligase complexes which play crucial roles in regulating response to JA and regulating plant gene expression during plant-pathogen interactions [37]. Mutants *coi*1-1 to *coi*1-14 were found to have enhanced resistance to *Pseudomonas syringae atropurpurea* [38]. Thus, the up-regulation of this gene could possibly reduce the resistance of oil palm roots to *G. boninense* due to suppression of JA-induced defence response. The decrease of JA in G-treated oil palm roots is further corroborated by the down-regulation of a JA-inducible gene encoding phenylalanine ammonia lyase (PAL). PAL is a key enzyme for the biosynthesis of cinnamic acid (CA) precursor which can be channeled to the biosynthetic pathways of monolignols, flavone/flavonoid, isoflavone phytoalexin, and SA. The down-regulation of an oil palm *PAL* gene (*EgPAL*) in G-treated oil palms was also supported by a previous study [21].

The accumulation of transcripts related to the methylation of SA to its activated form (methyl-SA) in G-treated oil palm suggests the involvement of methyl-SA in hormone signaling and possibly in systemic response in neighboring uninfected cells. True enough, the genes encoding NPR1 and NPR interactor which are involved in the signal transduction pathway that leads to SA-mediated systemic acquired resistance (SAR) [39], were also up-regulated in G-treated oil palm roots. The level of ET in G-treated oil palm tissues could be modulated by the expression of genes encoding ACC oxidase-like proteins. Different isoforms of ACC oxidases appear to be the principal targets whereby different gene sequences encoding this enzyme were up- and down-regulated in G-treated oil palm roots, complicated the deduction of the role of ET in G-treated oil palm roots. There could be “tug-of-war” between the pathogen and host to regulate cell death through ET in G-treated oil palm roots. ET was demonstrated to inhibit symptom development in necrotrophic pathogen infection but enhance the cell death caused by other type of pathogen infection [40]. Since *G. boninense* is a hemibiotroph, the regulation of ET in oil palm could be of central importance to determine the host resistance to this pathogen.

Our results suggested that the interplay of JA, SA and ET signaling pathways may partly determine the downstream molecular response in G-treated oil palm. SA accumulation was reported to increase the resistance of host plant to hemibiotrophic fungi but promote the susceptibility to necrotrophic pathogens [41, 42]. The accumulation of SA and SA signaling may negatively regulate the gene expression of ascorbate oxidase and ascorbate peroxidase which are involved in the scavenging of hydrogen peroxide [43] in G-treated oil palm roots. Hydrogen peroxide has been reported to be involved in HR which limits disease progression caused by biotroph but promotes the invasion of necrotroph. Since pathogenic *G. boninense* is a hemibiotroph, the modulation of cellular hydrogen peroxide in infected host cells could be critical in determining the disease tolerance/resistance of the host. In this study, a gene encoding hypersensitive-induced reaction (HIR) protein and two genes encoding autophagy related proteins were up-regulated; while a gene encoding defender against apoptotic death was down-regulated in G-treated oil palm roots. The oil palm HIR may have similar function as the rice HIR1 (OsHIR1) which can trigger hypersensitive cell death [44]. This may partly contribute to the susceptibility of oil palm roots to the necrotrophic *G. boninense*.

The downstream oil palm defense responses may involve the activation of various transcription factors (TFs) mainly AP2/ERE, bZIP, Zn finger, NAC, MYB and WRKY (Figs. 2 and 3) by concerted signals. As a result, various pathogenesis-related (PR) proteins were accumulated as evident by the up-regulation of their gene expression. The putative transcripts encoding PR proteins (including glucanases, chitinases, type 2 ribosome inactivating proteins, Bowman Birk serine protease inhibitor, type 2 proteinase inhibitor proteinases, cysteine and subtilisin proteinases), cell wall modifying enzymes such as pectinesterase and its inhibitor, pectate lyase, callose synthase, cellulose synthase and laccases, were up-regulated in G-treated oil palms. The production of PR proteins and cell wall modification (including callose deposition) are some of the defense mechanisms reported to be operating in host plants in response to pathogenic invasion [45]. Callose deposition causes blockage of plasmodesmata impeding cell-to cell movement of pathogens while methyl esterified cell wall pectin is less susceptible to hydrolysis of fungal polygalacturonases [46].

It is noted that many genes encoding xyloglucan endotransglucosylase/hydrolase and xyloglucan glycosyltransferases were down-and up-regulated, respectively, in the G-treated oil palm roots. Xylose was found to be able to enhance the production of fungal laccase which is a lignolytic enzyme [47]. Infected oil palm could possibly strengthen the cell wall by reducing the accumulation of xyloglucan degrading enzymes (thus reducing fungal laccase), by modifying xyloglucans in the host cell wall. The amount of lignin may also increase in G-treated oil palms due to the accumulation of transcripts related to monolignol biosynthesis especially trans-cinnamate 4-monooxygenase and caffeic acid 3-O-methyltransferase. As the first line of barrier to pathogen, cell wall and its biosynthesis and modification may play an important role in oil palm in its defense against *G. boninense*.

The up-regulation of genes encoding isoflavone 2’-hydroxylase and isoflavone 3’-hydroxylase in G-treated oil palms suggested an accumulation of unknown isoflavones. However, the down-regulation of a gene encoding isoflavone reductase in G-treated oil palms may dampen the accumulation of phytoalexin in G-treated oil palm roots. A rice isoflavone reductase-like gene, OsIRL, was demonstrated to be positively regulated by JA and negatively regulated by SA [48]. It is possible that the fungal elicitor from *G. boninense* could suppress JA and the JA-induced defence in G-treated oil palms including the down-regulation of isoflavone reductase. Furthermore, a high number of transcripts encoding sesquiterpene synthases, cytochrome P450s and a few other enzymes (in less number of transcripts) that are involved in terpene, sterol and steroid biosynthesis were also differentially expressed suggesting the accumulation of biochemical compounds in G-treated oil palms. A complex regulation of terpenoid pathway may lead to the production of sesquiterpenoid phytoalexins with fungitoxicity and also phytosterols that may restrict cell membrane permeability [49]. However, the types and roles of these secondary metabolites in oil palm defense await further elucidation.

### What are the expressed genes from *G. boninense* that are involved in colonization of oil palm roots?

As a white rot fungus, *G. boninense* is able to degrade and mineralize all components of plant cell walls, including the recalcitrant lignin. Transcriptome sequencing of G-treated oil palm roots also allowed us to have an overview of the fungal genes being expressed in G-treated oil palm roots, including genes encoding enzymes involved in lignin degradation (manganese peroxidase and laccases). Besides, there are also fungal transcripts encoding many glycoside hydrolases and glycosyltransferases from various families, carbohydrate binding modules, cellulases, pectinesterases, polysaccharide lyase etc., suggesting their active roles in degrading other plant cell wall components. Fungal transcripts encoding glyoxal oxidase which is required for filamentous growth and pathogenicity in *Ustilago maydis* [50] were also found among the fungal transcripts in G-treated oil palm roots. Glyoxal oxidase was also found to have a role in peroxide production for diverse oxidative reactions during wood decay [51].

Fungal gene sequences that are involved in ergosterol biosynthesis were found among transcripts in G-treated oil palms exclusively. As mentioned earlier, ergosterol which is prevalent in the membrane of *G. boninense* may be perceived by oil palm PRP as a PAMP which triggers PTI in the host plant. Ergosterol was shown to be able to trigger differential changes in the metabolome and variation in the biosynthesis of secondary metabolites in tobacco cells [49]. It also induces expression of genes encoding PR proteins and PAL [52]. In addition, immunomodulatory protein and allergens from *G. boninense* may also function in triggering host defense. The presence of a fungal gene encoding necrosis- and ethylene-inducing (NEP) 1 protein in G-treated oil palm roots is of particular interest as this protein has been found to be able to induce the formation of necrotic lesions and expression of genes encoding PR proteins, as well as to trigger the production of ROS in host plants [53]. NEP1 may cause phytotoxicity to oil palm roots through cytolysis and increased membrane permeability [54].

On the other hand, the presence of fungal transcripts encoding catalases, toxin detoxification proteins, and multidrug transporters, may assist the pathogen surviving the defense mechanism of host plants. Furthermore, two different fungal transcripts encoding SA hydroxylases were found in G-treated oil palm root and untreated roots respectively. The questions as to whether these enzymes were secreted into host cells and able to degrade host SA are unknown. A few transcripts encoding fungal elicitors that have been reported in other pathosystems were discovered among the fungal transcripts, however, their roles await further investigation. All in all, the gene products mentioned above may contribute to the virulence of *G. boninense*.

### How did the avirulent symbiont *T. harzianum* evade host defense?

Approximately 20 % and 37 % of the GOSlim terms that are enriched among the up- and down-regulated genes in G-treated oil palm roots, was found to be also enriched among those in oil palm roots treated with *T. harzianum*, respectively. This shows that a common set of oil palm genes were up-and down-regulated by both fungi irrespective of whether they are pathogenic or avirulent fungi. However, different individual genes categorized under the same GOSlim may play different roles, shaping the final response adapted to the challenging pathogens (Table 2).

In plants colonized by *Trichoderma* sp., the endophytic fungus was found to be limited to a few root cortical cell layers in plant roots [55]. *T. harzianum* was able to colonize oil palm roots without causing any lesions. Since the T-treated oil palm seedlings were asymptomatic, the host may be able to limit the colonization of *Trichoderma* by reinforcing cell wall and producing antimicrobial compounds and ROS [56–60]. This is further substantiated by the differential expression of genes encoding enzymes involved in the production of secondary metabolites (mainly alkaloids, glycoside and ketone with unknown functions), cell wall biosynthesis, cell wall modification and respiratory burst in T-treated oil palm roots. *T. harzianum* may remain accommodated by the host as an avirulent symbiont by suppressing plant defense through the secretion of still unknown fungal effectors [61].

The enzymes involved in the biosynthesis of ergosterol was absent in T-treated oil palm roots indicating that this fungal cell membrane sterol may not serve as a PAMP in T-treated oil palm roots that triggers the downstream defense response. Indeed, the ergosterol-inducible genes encoding sesquiterpene synthases [62] were found to be not differentially expressed in T-treated oil palm roots.

The interaction between the oil palm and *T. harzianum* must have been finely tuned with promising benefits to both partners [61]. The expression of three genes encoding ACC oxidases were found to be down-regulated, while another one was found to be up-regulated in T-treated oil palm roots compared to those in untreated oil palms. Since many of these genes were different from those encoding the same enzyme in G-treated oil palms, it is obvious that these genes were elegantly regulated by different signals. The genes encoding OPR and COI were down- and up-regulated in T-treated oil palm roots, respectively, indicating the suppression of JA biosynthesis and JA-mediated defense response in T-treated oil palm roots. It is not surprising since *T. harzianum* may not be causing necrosis or wounding to oil palm roots. The up-regulation of three genes encoding NPR1-like proteins suggests that an active SA signaling pathway is present in T-treated oil palms. The cross talks of hormones may decide the final defense response in the host, allowing the survival of *T. harzianum* but effectively containing its spread in plant host*.*

Although the genes encoding Rbohs were up-regulated and SA signaling could negatively regulate the gene expression of enzymes which are involved in the scavenging of hydrogen peroxide in T-treated oil palm roots (Fig. 2), the gene expression of two genes encoding defender against apoptotic death were up-regulated, suggesting that their gene products may serve as negative regulators of cell death [63] in T-treated oil palm roots. It is logical that *T. harzianum* could avoid cell death in host cells as one of its survival strategies in host plants.

Unlike G-treated oil palm roots, the gene encoding polygalacturonase was down-regulated in T-treated oil palm roots, implicating differences in cell wall modification mechanisms and possibly also the types of DAMP induced by the two fungi. On the other hand, fewer fungal genes encoding cell wall degrading/modification enzymes were expressed in the transcriptome of T-treated oil palm roots. Transcripts encoding laccases and peroxidases were not found among the fungal genes in T- treated oil palm roots. It is possibly important for the fungus to avoid from generating DAMPs that may trigger host defense system in its attempt to establish a symbiotic (or at least a neutral) relationship with its host plant. In addition, *T. harzianum* may avoid over accumulation of SA in the host plants by producing SA hydroxylase coinciding with the presence of a transcript encoding this enzyme among the fungal transcripts in the T-treated oil palm seedlings.

### What are the fungal genes from *T. harzianum* that are involved in biological control of *G. boninense*?

Several fungal elicitors from *T. virens* were reported to be able to activate plant basal immunity such as the ET-inducing xylanase [64], the proteinaceous non-enzymatic elicitor Sm1 [65, 66] and the 18 mer peptaibols [67]. Although these genes were not found among the genes in T-treated oil palm roots, we do not exclude the possibility that they are regulated by other mechanisms at translation or post-translational levels. The biological control mechanisms by *Trichoderma* spp. could also be achieved by direct effect of *Trichoderma* on pathogen through competition for space and nutrients, parasitisation of other fungi by producing antimicrobial compounds and antifungal enzymes (reviewed in [68]). Indeed, the genes encoding for phosphate transporters, hexose transporter, monosaccharide transporters and carbohydrate metabolism were found to be abundant among the genes in T-treated oil palm roots, in favor of better competition for nutrients. Fungal genes from *T. harzianum* that are potentially involved in parasitisation of *G. boninense* were unreported while those involved in the production of antimicrobial compounds and antifungal enzymes were not identified. Our results demonstrated that *T. harzianum* is more likely to strengthen the host plants by improving the nutrient status of host plants.

## Conclusions

Investigation on the transcriptomes of oil palm roots in response to *G. boninense* has enabled us to dissect the defense mechanisms involved in the establishment of BSR: 1. Ergosterol, fungal cell wall components, host cell wall components (degraded by fungal CWDEs) may elicit the defense responses of oil palm roots; 2. JA biosynthesis, signaling and JA-mediated defense response may be suppressed while the SA biosynthesis, signaling and SA-mediated defense could be activated in G-treated oil palm roots by the pathogen; 3. *G. boninense* and the host plant may compete to control the establishment of disease symptom through the transcriptional regulation of ET biosynthesis, hydrogen peroxide production and scavenging, apoptosis and autophagy; 4. Colonized oil palm roots may produce PR proteins and secondary metabolites that have antifungal activities against *G. boninense*; and 5. Colonized oil palm may strengthen its cell wall through cell wall modifying enzymes as its defense response.

The endophytic *T. harzianum* could possibly evade the defense response of oil palm roots by suppressing both JA and ET biosynthesis, signaling and defense responses and avoid establishment of cell-death. However, oil palm could limit the spread of this avirulent symbiotic fungus by producing tailored made secondary metabolites from different groups. Analyses of the transcriptome of oil palm roots colonized with *T. harzianum* shed light on the possible biocontrol mechanisms employed by *T. harzianum* against *G. boninense* which include improved nutrition status of host plants through mobilization of nutrients. The findings of this analysis enhance our understanding on the molecular interactions of *G. boninense* and oil palm, and also the biocontrol mechanisms involving *T. harzianum*, thus contributing to future formulations of better strategies to eradicate BSR.

## Methods

### Preparation of *Ganoderma boninense*-inoculated rubber wood blocks

Rubber wood blocks (6 cm x 6 cm x 12 cm each) were sterilized at 121 °C for 30 min before they were added with 100 ml potato sucrose agar (PSA) per rubber wood block and autoclaved. The autoclaved wood blocks were inoculated with *Ganoderma boninense* PER71 (Malaysian Palm Oil Board, Bangi, Malaysia) cultures that were grown on PSA at 28 °C for 7 d, and incubated in the dark at room temperature for 8 weeks. The fungal strain selected for this study is a reference strain which has been reported to be pathogenic to oil palm [12, 13, 15].

### Preparation of surface mulch and conidial suspension from *Trichoderma harzianum*

For the preparation of conidial suspension, *Trichoderma harzianum* Rifai strain T32 (Faculty of Science, Universiti Putra Malaysia, Serdang, Malaysia) was grown on potato dextrose agar (PDA; BD, France) at 25 °C for 7 d. The spores were then harvested and the concentration of conidia was determined using a haemocytometer (Neubauer, Marienfeld, Germany). To prepare surface mulch from *T. harzianum*, approximately 150 g palm pressed fibers (PPF) of empty fruit bunch (EFB) were rinsed with water and autoclaved at 121 °C for 45 min in a plastic bag. The autoclaved PPF and EFB were inoculated with 20 ml of *T. harzianum* conidial suspension containing 1–9 x 10^8^ spores/ml, sealed and incubated at 25 °C for two weeks in the dark.

### Treatments of oil palm seedlings

A total of 27 five-month-old oil palm *Elaeis guineensis* GH 500 Series (*Dura* x *Pisifera*) seedlings were purchased from Sime Darby Seeds and Agricultural Services Sdn. Bhd. (Banting, Malaysia) for the following treatments: 1. artificial inoculation with *G. boninense* (G-treatment); 2. artificial inoculation with *T. harzianum* (T-treatment); and as controls (untreated oil palm seedlings). The genotypes of these oil palm seedlings have not been reported to be tolerant against *Ganoderma* spp. In fact, previous studies have found them to be susceptible to *G. boninense* [21].

For oil palm seedlings in the G-treatment, the roots of each oil palm seedling were placed in contact with a rubber wood block inoculated with *G. boninense* and covered with a mix of top soil and sand in a ratio of 2:1. For oil palm seedlings in the T-treatment, 300 g of *T. harzianum* surface mulch was placed at the basal part of each seedling, and 500 ml conidial suspension of *T. harzianum* was applied directly to each seedling every fortnight. The seedlings were arranged in a complete randomized design in a green house. Three seedlings were harvested from respective treatments at 3, 6 and 12 week post inoculation (wpi) with three untreated oil palm seedlings as controls. The whole roots were collected for analysis.

### RNA preparation

Total RNA from the roots of oil palm was isolated using a modified CTAB method as described by Wang et al. [69] and treated with DNase I (New England Biolabs, Hitchin, UK). The quality and integrity of RNA were evaluated using a spectrophotometer (Eppendorf, Hamburg, Germany) and 2100 Bioanalyzer (Agilent Technologies, Santa Clara, CA, USA). The RNA samples of nine biological replicates from each treatment or control were pooled for RNA sequencing and qRT-PCR.

### Massive RNA sequencing and de novo assembly of RNA-seq

RNA sequencing was conducted on RNA samples from untreated, G-, and T-treated oil palm roots, with the Illumina technology (Illumina, San Diego, CA, USA). The mRNA-seq data have been deposited at European Nucleotide Archive (ENA) under the accession number PRJEB7252. The short reads obtained from the roots were converted to FASTQ format and analyzed with Fastxtool (http://hannonlab.cshl.edu/fastx_toolkit/) for quality check with a minimum Phred value of 20. To remove the sequences that have high identities to fungal sequences, the sequence reads were mapped to the genome sequences of *Ganoderma* sp. 10597 SS1, *T. harzianum* CBS226.95, *T. virens* Gv29-8 and *T. reesei* (downloaded from Department of Energy Joint Genome Institutes; JGI; http://genome.jgi-psf.org/), with Bowtie 0.12.7 (Langmead et al. 2009). After the removal of probable fungal sequences, *de novo* assembly of RNA-seq was performed with Velvet (version 1.1.05, [70]) and Oases assembler (version 0.1.22, [71]) at an optimized k-mer size for each data set. The assembled sequences from the root samples were then clustered with TGICL [72] and CAP3 [73] with a minimum overlap length of 100 bp and an identity at 97 % to form unigenes.

### Annotation of trancripts

The unigenes were translated into six reading frames and compared with non-redundant (nr) database at the National Center for Biotechnology Information (NCBI) with BLASTX [74]. Matches with *E*-values equal or less than 10^−5^ were treated as ‘significant matches’. Sequences with no hits or matches with *E-*values more than 10^−5^ were classified as ‘non-significant matches’. Unigenes with their most significant matches originating from bacteria, fungi, animal and other non-plant organisms, were separated from plant sequences. The unigenes of plant and fungal origins were mapped to Gene Ontology (GO) with Blast2GO [75] (www.blast2go.com/). GOSlim terms were assigned to each unigene.

### Identification of differentially expressed genes (DEGs)

The sequence reads from each sample (without the fungal sequences) were mapped to the unigenes originating from plants with Tophat 2.02 [76]. The number of reads that mapped to each assembled sequence was calculated using HTseq-count (http://www-huber.embl.de/users/anders/HTSeq/). DESeq [29] was used to identify differentially expressed genes (DEGs) between two samples. The variance was estimated by treating the samples being compared as if they were replicates of the same condition [29]. Genes that have a log_2_ [Number of reads in treated oil palm roots/Number of reads in untreated oil palm roots] ≥ |2.0| (corresponding to 4-fold or more for up- down-regulation, respectively) with a *P*-value ≤ 0.05, were defined as significant DEGs in the two samples being compared. These oil palm DEGs were matched to *Arabidopsis* coding sequences (CDS) TAIR10 build 29 (ftp://ftp.ensemblgenomes.org/pub/plants/release-29/fasta/arabidopsis_thaliana/cds/) by tblastx with a maximum *E*-values equal or less than 10^−5^, and displayed by MapMan software (http://mapman.gabipd.org/) [77].

### Enrichment of GO Slim terms among DEGs

The enrichment of GO Slim terms among the DEGs was tested with Fisher's Exact Test with Multiple Testing Correction of false discovery rate (FDR) [78]. GOSlim term with a FDR which is equal or less than 0.05 was considered to be significantly “enriched”. The enriched GO terms were summarized by REVIGO [79] by removing redundant GO terms and presented by Circos software [80].

### Verification of DEGs

Verification of DEGs was conducted using the same plant materials and RNA samples used for mRNA-seq. RNA treated with DNaseI (1.2 μg) was reverse-transcribed with Affinity Script QPCR cDNA Synthesis Kit (Stratagene, La Jolla, CA, USA) according to the instructions of the manufacturer. The genes and primers used for real-time qRT-PCR are listed in Additional file1: Table S4. Real-time quantitative reverse transcription (qRT)-PCR was performed in BIO-RAD iQ5 iCycler Thermal Cycler (Bio-Rad, Berkeley, CA, USA) in quadruplicates. Each PCR reaction (20 μl) contained 200 ng of the first strand cDNA, 200 nM forward and reverse primers, 30nM reference dye, and 1× master mix from Brilliant III Ultra-Fast SYBR® Green QPCR Master Mix (Stratagene, La Jolla, CA, USA). The PCR condition was 95 °C for 3 min; 40 cycles of 95 °C for 5 s and 60 °C for 10 s. Two endogenous controls encoding actin and cyclophilin from oil palm were amplified in parallel for normalization and quantification. The amplification efficiency was estimated according to the equation: E=[(10^-1/y^)-1]×100 [81]. The relative transcript levels were calculated based on a comparative C_T_ method using multiple reference genes for normalization [82].

## Availability of supporting data

The raw reads for this project have been deposited in the European Nucleotide Archive (ENA) under the accession number PRJEB7252.

## Additional file

Additional file 1: Table S1. DEGs in G-treated oil palm roots compared to untreated roots. **Table S2.** DEGs in T-treated oil palm roots compared to untreated roots. **Table S3.** Fungal transcripts in oil palm transcriptomes. **Table S4.** List of qRT-PCR primers used for verification of DEGs. (XLSX 833 kb)

## Abbreviations

ACC-1: Aminocyclopropane-1-carboxylate
ARF: ADP-ribosylation factor
Avr: Avirulence
BP: Biological process
BSR: Basal stem rot
CDPK: Calcium dependent protein kinase
CDS: Coding sequences
CWDE: Cell wall degrading enzymes
CC: Cellular compartment
CA: Cinnamic acid
cfu: Colonies per unit
COI: Coronatine-insensitive 1
DAMP: Damage-associated molecular pattern
DEGs: Differentially expressed genes
ETI: Effector-triggered immunity
EFB: Empty fruit bunch
ET: Ethylene
EIN: ET-insensitive
FDR: False discovery rate
GO: Gene Ontology
HR: Hypersensitive reaction
HIR: Hypersensitive-induced reaction
JA: Jasmonate
MAPK: Mitogen associated protein kinase
MF: Molecular function
MI: Myo-inositol
NCBI: National Center for Biotechnology Information
NEP: Necrosis- and ethylene-inducing protein
OPDA: 12-Oxophytodienoate
PPF: Palm pressed fibers
PTI: PAMP-triggered immunity
PAMP: Pathogen-associated molecular pattern
PR: Pathogenesis related
PRR: Pattern recognition receptor
PAL: Phenylalanine ammonia lyase
PDA: Potato dextrose agar
PSA: Potato sucrose agar
qRT: Quantitative reverse-transcription
ROS: Reactive oxygen species
RLK: Receptor-like kinase
RLP: Receptor-like protein
R protein: Resistance protein
Rboh: Respiratory burst oxidase
RIN4: RPM1-interacting protein 4
SA: Salicylate
SOD: Superoxide dismutase
SAR: Systemic acquired resistance
TF: Transcription factor
Wpi: Weeks post inoculation

## References

[1] Singh G (1991). *Ganoderma* – the scourge of oil palms in the coastal areas. Planter.

[2] Turner P (1981). Oil Palm Diseases and Disorders.

[3] Paterson RRM (2007). *Ganoderma* disease of oil palm-A white rot perspective necessary for integrated control. Crop Prot.

[4] Chung GF (2011). Management of *Ganoderma* diseases in oil palm plantations. Planter.

[5] Mohd As’wad AW, Sariah M, Paterson RRM, Zainal Abidin MA, Lima N (2011). Ergosterol analyses of oil palm seedlings and plants infected with *Ganoderma*. Crop Prot.

[6] Bridge P, Ogrady E, Pilotti C, Sanderson F (2000). Development of Molecular Diagnostics for the Detection of Ganoderma Isolates Pathogenic to Oil Palm.

[7] Idris A, Yamaoka M, Hayakawa S, Basri M, Noorhasimah I, Ariffin D: PCR technique for detection of *Ganoderma. MPOB Inf Ser* 2003

[8] Utomo C, Niepold F (2000). Development of diagnostic methods for detecting *Ganoderma*-infected oil palms. J Phytopathol.

[9] 9 Breton F, Hasan Y, Hariadi S, Lubis Z, de Franqueville H: Characterization of parameters for the development of an early screening test for basal stem rot tolerance in oil palm progenies. *J Oil Palm Res* 2006, Special is:24–36.

[10] Soepena H, Purba R, Pawirosukarto S (2000). A Control Strategy for Basal Stem Rot (Ganoderma) on Oil Palm.

[11] Nur Ain Izzati MZ, Abdullah F (2008). Disease suppression in *Ganoderma*-infected oil palm seedlings treated with *Trichoderma harzianum*. Plant Prot Sci.

[12] Siddiquee S, Yusuf UK, Hossain K, Jahan S (2009). In vitro studies on the potential Trichoderma harzianum for antagonistic properties against *Ganoderma boninense*. J Food Agric Env.

[13] Shamala S, Faridah A, Zainal A, Umi K (2008). Efficacy of single and mixed treatments of *Trichoderma harzianum* as biocontrol agents of *Ganoderma* basal stem rot in oil palm. J Oil Palm Res.

[14] Durand-Gasselin T, Asmady H, Flori A, Jacquemard JC, Hayun Z, Breton F (2005). Possible sources of genetic resistance in oil palm (*Elaeis guineensis* Jacq.) to basal stem rot caused by *Ganoderma boninense*-prospects for future breeding. Mycopathologia.

[15] Idris A, Kushairi A, Ismail S, Ariffin D (2004). Selection for partial resistance in oil palm progenies to *Ganoderma* basal stem rot. J Oil Palm Res.

[16] Naher L, Ho CL, Tan SG, Yusuf UK, Abdullah F (2011). Cloning of transcripts encoding chitinases from *Elaeis guineensis* Jacq. and their expression profiles in response to fungal infections. Physiol Mol Plant Pathol.

[17] Tan YC, Yeoh KA, Wong MY, Ho CL (2013). Expression profiles of putative defence-related proteins in oil palm (*Elaeis guineensis*) colonized by *Ganoderma boninense*. J Plant Physiol.

[18] Yeoh KA, Othman A, Meon S, Abdullah F, Ho CL (2012). Sequence analysis and gene expression of putative exo- and endo-glucanases from oil palm (*Elaeis guineensis*) during fungal infection. J Plant Physiol.

[19] Yeoh KA, Othman A, Meon S, Abdullah F, Ho CL (2013). Sequence analysis and gene expression of putative oil palm chitinase and chitinase-like proteins in response to colonization of *Ganoderma boninense* and *Trichoderma harzianum*. Mol Biol Rep.

[20] Alizadeh F, Abdullah SNA, Khodavandi A, Abdullah F, Yusuf UK, Chong PP (2011). Differential expression of oil palm pathology genes during interactions with *Ganoderma boninense* and *Trichoderma harzianum*. J Plant Physiol.

[21] Tee SS, Tan YC, Abdullah F, Ong-Abdullah M, Ho CL (2013). Transcriptome of oil palm (*Elaeis guineensis* Jacq.) roots treated with *Ganoderma boninense*. Tree Genet Genomes.

[22] Ariani A, Di Baccio D, Romeo S, Lombardi L, Andreucci A, Lux A (2015). RNA sequencing of *Populus x canadensis* roots identifies key molecular mechanisms underlying physiological adaption to excess zinc. PLoS ONE.

[23] Jongeneel CV, Delorenzi M, Iseli C, Zhou D, Haudenschild CD, Khrebtukova I (2005). An atlas of human gene expression from massively parallel signature sequencing (MPSS). Genome Res.

[24] Huang W, Khatib H (2010). Comparison of transcriptomic landscapes of bovine embryos using RNA-Seq. BMC Genomics.

[25] Md-Mustafa ND, Khalid N, Gao H, Peng Z, Alimin MF, Bujang N (2014). Transcriptome profiling shows gene regulation patterns in a flavonoid pathway in response to exogenous phenylalanine in *Boesenbergia rotunda* cell culture. BMC Genomics.

[26] Ye M, Chen Z, Su X, Ji L, Wang J, Liao W (2014). Study of seed hair growth in *Populus tomentosa*, an important character of female floral bud development. BMC Genomics.

[27] Zenoni S, Ferrarini A, Giacomelli E, Xumerle L, Fasoli M, Malerba G (2010). Characterization of transcriptional complexity during berry development in Vitis vinifera using RNA-Seq. Plant Physiol.

[28] Zhang J, Wu K, Zeng S, da Silva JAT, Zhao X, Tian C-E (2013). Transcriptome analysis of *Cymbidium sinense* and its application to the identification of genes associated with floral development. BMC Genomics.

[29] Anders S, Huber W (2010). Differential expression analysis for sequence count data. Genome Biol.

[30] Doehlemann G, Wahl R, Horst RJ, Voll LM, Usadel B, Poree F (2008). Reprogramming a maize plant: Transcriptional and metabolic changes induced by the fungal biotroph *Ustilago maydis*. Plant J.

[31] De Lorenzo G, Cervone F, Bellincampi D, Caprari C, Clark A, Desiderio A (1994). Polygalacturonase, PGIP and oligogalacturonides in cell-cell communication. Biochem Soc Trans.

[32] Dangl JL, Jones JD (2001). Plant pathogens and integrated defence responses to infection. Nature.

[33] Staal J, Kaliff M, Dewaele E, Persson M, Dixelius C (2008). RLM3, a TIR domain encoding gene involved in broad-range immunity of *Arabidopsis* to necrotrophic fungal pathogens. Plant J.

[34] Liu J, Elmore JM, Fuglsang AT, Palmgren MG, Staskawicz BJ, Coaker G (2009). RIN4 functions with plasma membrane H^+^-ATPases to regulate stomatal apertures during pathogen attack. PLoS Biol.

[35] De-Jong AJ, Yakimova ET, Kapchina VM, Woltering EJ (2002). A critical role for ethylene in hydrogen peroxide release during programmed cell death in tomato suspension cells. Planta.

[36] Croft K, Juttner F, Slusarenko AJ (1993). Volatile products of the lipoxygenase pathway Evolved from *Phaseolus vulgaris* (L.) leaves inoculated with *Pseudomonas syringae* pv *phaseolicola*. Plant Physiol.

[37] Xu L, Liu F, Lechner E, Genschik P, Crosby WL, Ma H (2002). The SCF(COI1) ubiquitin-ligase complexes are required for jasmonate response in *Arabidopsis*. Plant Cell.

[38] Xie DX, Feys BF, James S, Nieto-Rostro M, Turner JG (1998). COI1: an *Arabidopsis* gene required for jasmonate-regulated defense and fertility. Science.

[39] Feys BJ, Parker JE (2000). Interplay of signaling pathways in plant disease resistance. Trends Genet.

[40] Wang KL, Li H, Ecker JR (2002). Ethylene biosynthesis and signaling networks. Plant Cell.

[41] Veronese P, Chen X, Bluhm B, Salmeron J, Dietrich R, Mengiste T (2004). The BOS loci of *Arabidopsis* are required for resistance to *Botrytis cinerea* infection. Plant J.

[42] Veronese P, Nakagami H, Bluhm B, Abuqamar S, Chen X, Salmeron J (2006). The membrane-anchored BOTRYTIS-INDUCED KINASE1 plays distinct roles in *Arabidopsis* resistance to necrotrophic and biotrophic pathogens. Plant Cell.

[43] Mittler R (2002). Oxidative stress, antioxidants and stress tolerance. Trends Plant Sci.

[44] Zhou L, Cheung M-Y, Li M-W, Fu Y, Sun Z, Sun S-M (2010). Rice hypersensitive induced reaction protein 1 (OsHIR1) associates with plasma membrane and triggers hypersensitive cell death. BMC Plant Biol.

[45] Hammond-Kosack KE, Jones JD (1996). Resistance gene-dependent plant defense responses. Plant Cell.

[46] Lionetti V, Raiola A, Camardella L, Giovane A, Obel N, Pauly M (2007). Overexpression of pectin methylesterase inhibitors in *Arabidopsis* restricts fungal infection by *Botrytis cinerea*. Plant Physiol.

[47] Songulashvili G, Elisashvili V, Wasser S, Hadar Y, Nevo E (2008). Effect of the carbon source and inoculum preparation method on laccase and manganese peroxidase production in submerged cultivation by the medicinal mushroom *Ganoderma lucidum* (W Curt: Fr) P Karst (Aphyllophoromycetideae). Int J Med Mushrooms.

[48] Kim ST, Cho KS, Kim SG, Kang SY, Kang KY (2003). A rice isoflavone reductase-like gene, OsIRL, is induced by rice blast fungal elicitor. Mol Cells.

[49] Tugizimana F, Steenkamp PA, Piater LA, Dubery IA (2014). Multi-platform metabolomic analyses of ergosterol-induced dynamic changes in *Nicotiana tabacum* cells. PLoS One.

[50] Leuthner B, Aichinger C, Oehmen E, Koopmann E, Müller O, Müller P (2005). A H_2_O_2_-producing glyoxal oxidase is required for filamentous growth and pathogenicity in *Ustilago maydis*. Mol Genet Genomics.

[51] Kersten P, Cullen D (2014). Copper radical oxidases and related extracellular oxidoreductases of wood-decay Agaricomycetes. Fungal Genet Biol.

[52] Lochman J, Mikes V (2006). Ergosterol treatment leads to the expression of a specific set of defence-related genes in tobacco. Plant Mol Biol.

[53] Wang JY, Cai Y, Gou JY, Mao YB, Xu YH, Jiang WH (2004). VdNEP, an elicitor from *Verticillium dahliae*, induces cotton plant wilting. Appl Environ Microbiol.

[54] Ottmann C, Luberacki B, Küfner I, Koch W, Brunner F, Weyand M (2009). A common toxin fold mediates microbial attack and plant defense. Proc Natl Acad Sci U S A.

[55] Harman GE, Howell CR, Viterbo A, Chet I, Lorito M (2004). *Trichoderma* species-opportunistic, avirulent plant symbionts. Nat Rev Microbiol.

[56] Yedidia I, Benhamou N, Chet I (1999). Induction of defense responses in cucumber plants (*Cucumis sativus* L.) by the biocontrol agent *Trichoderma harzianum*. Appl Environ Microbiol.

[57] Yedidia I, Benhamou N, Kapulnik Y, Chet I (2000). Induction and accumulation of PR proteins activity during early stages of root colonization by the mycoparasite *Trichoderma harzianum* strain T-203. Plant Physiol Biochem.

[58] Chacón MR, Rodríguez-Galán O, Benítez T, Sousa S, Rey M, Llobell A (2007). Microscopic and transcriptome analyses of early colonization of tomato roots by *Trichoderma harzianum*. Int Microbiol.

[59] Contreras-Cornejo HA, Macías-Rodríguez L, Beltrán-Peña E, Herrera-Estrella A, López-Bucio J (2011). *Trichoderma*-induced plant immunity likely involves both hormonal- and camalexin-dependent mechanisms in *Arabidopsis thaliana* and confers resistance against necrotrophic fungi *Botrytis cinerea*. Plant Signal Behav.

[60] Salas-Marina MA, Silva-Flores MA, Uresti-Rivera EE, Castro-Longoria E, Herrera-Estrella A, Casas-Flores S (2011). Colonization of *Arabidopsis* roots by *Trichoderma atroviride* promotes growth and enhances systemic disease resistance through jasmonic acid/ethylene and salicylic acid pathways. Eur J Plant Pathol.

[61] Martínez-Medina A, Fernández I, Sánchez-Guzmán MJ, Jung SC, Pascual JA, Pozo MJ (2013). Deciphering the hormonal signalling network behind the systemic resistance induced by *Trichoderma harzianum* in tomato. Front Plant Sci.

[62] Tugizimana F, Steenkamp PA, Piater LA, Dubery IA (2012). Ergosterol-induced sesquiterpenoid synthesis in tobacco cells. Molecules.

[63] Kelleher DJ, Gilmore R (1997). DAD1, the defender against apoptotic cell death, is a subunit of the mammalian oligosaccharyltransferase. Proc Natl Acad Sci U S A.

[64] Hanson LE, Howell CR (2004). Elicitors of plant defense responses from biocontrol strains of *Trichoderma viren*. Phytopathology.

[65] Djonović S, Pozo MJ, Dangott LJ, Howell CR, Kenerley CM (2006). Sm1, a proteinaceous elicitor secreted by the biocontrol fungus *Trichoderma virens* induces plant defense responses and systemic resistance. Mol Plant Microbe Interact.

[66] Djonovic S, Vargas WA, Kolomiets MV, Horndeski M, Wiest A, Kenerley CM (2007). A proteinaceous elicitor Sm1 from the beneficial fungus *Trichoderma vire*ns is required for induced systemic resistance in maize. Plant Physiol.

[67] Viterbo A, Wiest A, Brotman Y, Chet I, Kenerley C (2007). The 18mer peptaibols from *Trichoderma virens* elicit plant defence responses. Mol Plant Pathol.

[68] Vinale F, Sivasithamparam K, Ghisalberti EL, Marra R, Woo SL, Lorito M (2008). *Trichoderma*-plant-pathogen interactions. Soil Biol Biochem.

[69] Wang T, Zhang N, Du L (2005). Isolation of RNA of high quality and yield from *Ginkgo biloba* leaves. Biotechnol Lett.

[70] Zerbino DR, Birney E (2008). Velvet: Algorithms for de novo short read assembly using de Bruijn graphs. Genome Res.

[71] Schulz MH, Zerbino DR, Vingron M, Birney E (2012). Oases: Robust de novo RNA-seq assembly across the dynamic range of expression levels. Bioinformatics.

[72] Pertea G, Huang X, Liang F, Antonescu V, Sultana R, Karamycheva S (2003). TIGR gene indices clustering tools (TGICL): A software system for fast clustering of large EST datasets. Bioinformatics.

[73] Huang X, Madan A (1999). CAP3: A DNA sequence assembly program. Genome Res.

[74] Altschul SF, Madden TL, Schäffer AA, Zhang J, Zhang Z, Miller W (1997). Gapped BLAST and PSI-BLAST: A new generation of protein database search programs. Nucleic Acids Res.

[75] Conesa A, Götz S, García-Gómez JM, Terol J, Talón M, Robles M (2005). Blast2GO: A universal tool for annotation, visualization and analysis in functional genomics research. Bioinformatics.

[76] Trapnell C, Pachter L, Salzberg SL (2009). TopHat: Discovering splice junctions with RNA-Seq. Bioinformatics.

[77] Thimm O, Blaesing O, Gibon Y, Nagel A, Meyer S, Krüger P (2004). MAPMAN: a user-driven tool to display genomics data sets onto diagrams of metabolic pathways and other biological processes. Plant J.

[78] Benjamini Y, Hochberg Y (1995). Controlling the false discovery rate: a practical and powerful approach to multiple testing. J R Statist Soc B.

[79] Supek F, Bošnjak M, Škunca N, Šmuc T (2011). Revigo summarizes and visualizes long lists of gene ontology terms. PLoS One.

[80] Krzywinski MI, Schein JE, Birol I, Connors J, Gascoyne R, Horsman D (2009). Circos: An information aesthetic for comparative genomics. Genome Res.

[81] Rasmussen R, Meuer S, Wittwer C, Nakagawara K (2001). Quantification on the light cycler. Rapid Cycle Real-time PCR Methods Appl.

[82] Hellemans J, Mortier G, De Paepe A, Speleman F, Vandesompele J (2007). qBase relative quantification framework and software for management and automated analysis of real-time quantitative PCR data. Genome Biol.

